# MMP-2 release and activation in ovarian carcinoma: the role of fibroblasts

**DOI:** 10.1038/sj.bjc.6690357

**Published:** 1999-05-01

**Authors:** R S Boyd, F R Balkwill

**Affiliations:** Imperial Cancer Research Fund, Lincoln's Inn Fields, London WC2A 3PX, UK

**Keywords:** ovarian carcinoma, MMP-2, TIMP-2, collagen, fibroblasts

## Abstract

The matrix metalloproteinase MMP-2 is up-regulated in epithelial cancers and its mRNA localizes to stromal fibroblasts. In this paper we show that co-culture of ovarian carcinoma cells with fibroblasts resulted in an enhanced release of proMMP-2 and TIMP-2 into the culture medium. Cell–cell interaction was a major factor in this response and carcinoma cells stimulated proMMP-2 release from fibroblasts but not vice versa. Collagen I, in a dose-dependent fashion, induced activation of proMMP-2 by tumour-derived, but not normal, fibroblasts. Antibody to β_1_ integrin also induced proMMP-2 activation by tumour-derived fibroblasts. The activation involved the processing of proMMP-2 by a membrane-bound metalloproteinase. We propose that, in the ovarian tumour microenvironment, interaction between tumour cells and fibroblasts may enhance fibroblast production of the proMMP-2 and TIMP-2. Collagen I, also present in the ovarian tumours, then induces these fibroblasts to activate proMMP-2 even in the presence of TIMP-2. This active MMP-2 can associate with the cell surface of tumour cells and fibroblasts and is used in the processes of tissue remodelling and invasion. © 1999 Cancer Research Campaign


*British Journal of Cancer* (1999) **80**(3/4), 315–321
© 1999 Cancer Research Campaign
Article no. bjoc.1998.0357

### MMP-2 release and activation in ovarian carcinoma: the role of fibroblasts

**RS Boyd and FR Balkwill**

Imperial Cancer Research Fund, Lincoln's Inn Fields, London WC2A 3PX, UK

**Summary** The matrix metalloproteinase MMP-2 is up-regulated in epithelial cancers and its mRNA localizes to stromal fibroblasts. In this paper we show that co-culture of ovarian carcinoma cells with fibroblasts resulted in an enhanced release of proMMP-2 and TIMP-2 into the culture medium. Cell–cell interaction was a major factor in this response and carcinoma cells stimulated proMMP-2 release from fibroblasts but not vice versa. Collagen I, in a dose-dependent fashion, induced activation of proMMP-2 by tumour-derived, but not normal, fibroblasts. Antibody to $\beta_1$ integrin also induced proMMP-2 activation by tumour-derived fibroblasts. The activation involved the processing of proMMP-2 by a membrane-bound metalloproteinase. We propose that, in the ovarian tumour microenvironment, interaction between tumour cells and fibroblasts may enhance fibroblast production of the proMMP-2 and TIMP-2. Collagen I, also present in the ovarian tumours, then induces these fibroblasts to activate proMMP-2 even in the presence of TIMP-2. This active MMP-2 can associate with the cell surface of tumour cells and fibroblasts and is used in the processes of tissue remodelling and invasion.

**Keywords:** ovarian carcinoma; MMP-2; TIMP-2; collagen; fibroblasts

Matrix metalloproteinases (MMPs) are involved in the degradation of the extracellular matrix, a central element of tumour invasion and metastasis (Birkedal-Hansen, 1995). One of these enzymes, MMP-2, is able to degrade type IV collagen, which is a major component of the basement membrane (Liotta et al, 1991). This enzyme can also modulate carcinoma cell adhesion (Ray and Stetler-Stevenson, 1995).

MMP-2 is released in a pro-form which can be activated by membrane-bound metalloproteinases, MT-MMP-1, -2 and -3 (Sato et al, 1994; Kolenbrock et al, 1997; Shofuda et al, 1997) which themselves require prior activation (Lohi et al, 1996). The active forms of these membrane metalloproteinases cleave the 72 kDa pro-form of MMP-2 at a specific amino acid residue generating an active 64 kDa form (Strongin et al, 1995). The 64 kDa form of MMP-2 can be further processed to a 62 kDa form by autocatalysis (Stetler-Stevenson et al, 1989*a*; Atkinson et al, 1995) or the plasminogen activator (PA)/plasmin system (Baramova et al, 1997). A number of naturally occurring MMP inhibitors, TIMPs 1–4, are also found in tissues (Stetler-Stevenson et al, 1989*b*; Greene et al, 1996). The activation of proMMP-2 by active MT-MMPs is blocked by the tissue inhibitor TIMP-2 (Atkinson et al, 1995; Strongin et al, 1995). Paradoxically TIMP-2 is also necessary for the formation of the MT-MMP1 complex which activates MMP-2 (Strongin et al, 1995). The tissue inhibitors TIMPs 1–4 also block the activity of MMP-2 (Stetler-Stevenson et al, 1989*b*; Greene et al, 1996).

Previous studies have identified the presence of proMMP-2 and its active form in ovarian cancer specimens (Naylor et al, 1994; Garzetti et al, 1995; Tamakoshi et al, 1995). One study has found no association between the level of latent or activated MMP-2 and the grade of ovarian carcinoma, although all of the carcinomas investigated contained latent and active forms of MMP-2 (Naylor et al, 1994).

MMP-2 may be involved in two different aspects of ovarian carcinoma spread. Ovarian tumour cells could use MMP-2 to detach from surface epithelia and migrate into the peritoneal cavity (Niedbala et al, 1987), but they may also use MMP-2 to invade through basement membrane into the ovarian stroma (Campo et al, 1992; Kuwashima et al, 1995).

Fibroblasts at the invasive edge of ovarian tumours show a striking increase in mRNA for MMP-2 and TIMP-2 (Campo et al, 1992; Autio-Harminen et al, 1993; Naylor et al, 1994). While ovarian tumour cells in tumour tissue do not contain MMP-2 or TIMP-2 mRNA, latent and activated MMP-2 protein can be detected at their surface (Afzal et al, 1996). As it is possible that tumour cell lines could utilize fibroblast MMP-2 for invasion (Fishman et al, 1997), interactions between stromal cells and tumour cells may play an important part in ovarian carcinoma pathology in vivo. In this paper we report that fibroblast–carcinoma cell interactions in vitro result in the stimulation of proMMP-2 and TIMP-2 release by fibroblasts. We also describe a mechanism by which collagen I can induce proMMP-2 activation by tumour-derived, but not normal, fibroblasts.

*Received 18 March 1998*
*Revised 21 October 1998*
*Accepted 22 October 1998*

*Correspondence to:* FR Balkwill

### MATERIALS AND METHODS

### Materials

ProMMP-2 and antisera against MMP-2 or TIMP-2 were obtained from TCS Biologicals (Buckingham, UK). Anti-EMMPRIN antibody was a kind gift from Dr Bryan P Toole, Tufts University School of Medicine (Boston, MA, USA). The anti-$\beta_1$ integrin antibody was from Gibco (Paisley, UK); rat tail collagen I from Beckton-Dickinson, Bedford, MA, USA and ECL™ reagent was obtained from Amersham (Buckinghamshire, UK). The monoclonal antibodies against MMP-2, MT-MMP-1 or TIMP-2 were purchased from Oncogene Research Products (Cambridge, MA, USA). BB2116 is a synthetic hydroxamate MMP inhibitor which was obtained from British Biotech Pharmaceuticals (Oxford, UK). It was dissolved in dimethyl suphoxide as a 100 mM solution and diluted in phosphate-buffered saline (PBS) to a concentration of 30 μM. The cDNA synthesis 'Ready to Go Kit' was purchased from Pharmacia, (Madison, WI, USA). The PEO1 and PEO14 cells were kindly provided by Dr S Langdon (ICRF, Edinburgh Medical Oncology Unit, UK). SKOV3 cells were obtained from American Type Culture Collection (Rockville, MD, USA).

The human dermal fibroblasts were a gift from Simon Broad (ICRF, London, UK) and the human foreskin fibroblasts were provided by Central Cell Services (ICRF, London, UK). Ovarian tumour-derived fibroblasts were obtained by culture of tumour tissue explants (Noel et al, 1992) and were characterized using collagen I as a positive marker. The polymerase chain reaction (PCR) primers were MT-MMP-1: fCACTGCCTACGAGAGGAAGG, rTGAATGACCCTCTGGGAGAC; MT-MMP-2: fCGTGTCCTGCTTTACTGCAA, rCTCCAACTGGGCAAAGAGAG; MT-MMP-3: fCAGGGTGATGGATGGATGGATACC, rCCTTGAGGATGGATCTTGGA; MT-MMP-4: fACGAGGTCTGCTCATGCA, rCAGGGAGAGGTCATGTTGGT. Other reagents used were supplied by Sigma Chemicals Co (Poole, UK).

### Methods

### *Cell culture and experiments*

PEO1 and PEO14 cells were cultured in RPMI-1640 and 10% fetal calf serum. SKOV3 cells and fibroblasts were cultured in Dulbecco's modified Eagle medium (DMEM) and 10% fetal calf serum. The human dermal and foreskin fibroblasts were used between passages 5 and 10. In co-culture experiments, a mixture of $2.5 \times 10^4$ fibroblasts and $12.5 \times 10^4$ carcinoma cells was plated in 24-well plates (1.88 $cm^2$ per well) overnight at 37°C and 5% carbon dioxide. Cells were washed four times with 1 ml of serum-free medium and then incubated with 0.5 ml of medium +/– agents for 48 h at 37°C. The medium was then removed and frozen at –20°C. In some experiments, cells were fixed by incubation for 5 min with ice cold 80% methanol. Fixed cells were washed three times with medium before fibroblasts or carcinoma cells were added.

### *Quantitative gelatin zymography*

The methods used here are described in Leber and Balkwill (1997). Briefly, medium was treated with sample buffer and loaded onto sodium dodecyl sulphate (SDS)-polyacrylamide gels containing 0.12% gelatin. After electrophoresis the gel was incubated in 2.5% Triton X-100 for 1 h, low-salt collagenase buffer for 18 h at 37°C and then simultaneously stained and de-stained. The resulting gel was scanned using the Adobe Photoshop 4.0 software package. The NIH 1.58 software package was used to quantitate changes in gelatin concentration. The gelatinolytic activity of unknown samples was calibrated relative to a fixed amount of standard proMMP-2.

### *Triton X-114 extraction of proteins*

Hydrophobic proteins were separated from aqueous proteins by Triton X-114 at 37°C (Bordier, 1981). A total of $2 \times 10^6$ fibroblasts per 25 $cm^2$ well were washed four times in ice cold serum-free medium and 1 ml of ice cold 1.5% Triton X-114 TBS (50 mM Tris-HCl, pH 7.4, 150 mM sodium chloride, 2 mM $Mg^{2+}$, 5 mM $Ca^{2+}$) was added for 1 h at 4°C. Cells were scraped using a rubber policeman and the detergent mixture was spun at 13 000 ***g***. The supernatant was incubated at 37°C for 5 min and centrifuged 13 000 ***g*** for 2 min. The lower detergent phase, containing hydrophobic protein, was separated from the upper aqueous phase, containing hydrophilic proteins. The detergent phase was subjected to three more extractions. The aqueous and detergent phases were analysed for gelatinase activity as described (Lewalle et al, 1995).

### *ProMMP-2 processing by Triton X-114 membrane protein extracts*

In order to detect proMMP-2 processing activity by fibroblast membranes, fibroblasts ($2 \times 10^6$) were treated with Triton X-114 and 50 μl detergent extract was mixed with 50 μl of TBS, containing 0.02% sodium azide and proMMP-2 (10 ng). ProMMP-2 was also incubated with detergent alone as a control. The reaction mixture was incubated for 48 h at 37°C and the aqueous phase was analysed by gelatin zymography (Lewalle et al, 1995).

### *Western blotting*

Protein in supernatant from the co-culture experiments was precipitated with acetone, run on an SDS-polyacrylamide gel and blotted onto a nitrocellulose membrane. The membrane was blocked by 5% milk powder and incubated with specific anti-TIMP-2 or anti-MMP-2 antisera. The blot was treated with HRP-linked mouse or rabbit IgG and analysed using the ECL™ system. In some cases, $0.5 \times 10^6$ carcinoma cells or fibroblasts were washed and treated with 1.0 ml of 2.5% v/v Triton X-114 for 1 h at 4°C. The protein was extracted from the detergent using chloroform–methanol (Bordier, 1981) and analysed as described above.

### *Reverse transcription (RT)-PCR*

RNA was extracted from cells using the TRI™-reagent, digested by DNAse and converted to cDNA using a Pharmacia 'Ready To Go Kit'. cDNA was amplified using specific sets of primers and conditions (Liabakk et al, 1996).

### RESULTS

### MMP-2 and TIMP-2 release

Studies of ovarian carcinoma tissue have shown that it consists of nests of tumour cells surrounded by a stroma of fibroblast-like cells (Naylor et al, 1994). Co-culture of dermal fibroblasts and ovarian carcinoma cell lines resulted in similar structures to those seen in vivo with small islands of tumour cells surrounded by sheets of fibroblasts (Figure 1).

We used dermal fibroblasts to determine if co-culture of normal fibroblasts with tumour cells could enhance proMMP-2 release into the culture medium. When cultured alone, dermal fibroblasts released proMMP-2 as measured by zymography (Figure 2A). Ovarian carcinoma cell lines also released small amounts of proMMP-2 which varied with cell line (Figure 2A). In co-culture experiments with dermal fibroblasts and the ovarian tumour cell lines PEO1, PEO14 or SKOV3, a ratio of 1:5 was chosen because at the leading edge of tumour tissue there is an excess of tumour cells to

 © Cancer Research Campaign 1999

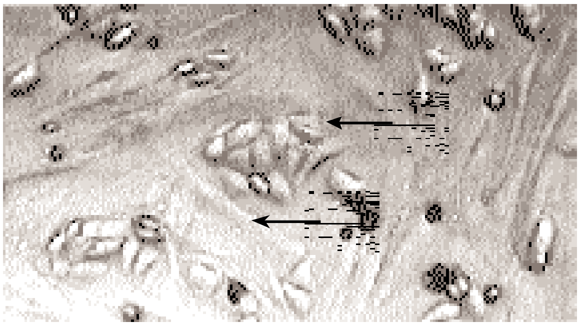

**Figure 1** Phase contrast microscopy of a co-culture of PEO1 cells and dermal fibroblasts. A total of $2.5 \times 10^4$ fibroblasts and $12.5 \times 10^4$ PEO1 cells were mixed and plated overnight in medium + serum. The cells were then washed three times and incubated for 48 h in serum-free medium. T = PEO1 cells, F = dermal fibroblasts fibroblasts (Naylor et al, 1994). A two- to eightfold enhanced release of proMMP-2 was found in such co-cultures (Figure 2A). This enhancement of proMMP-2 release during co-culture was quantified and found to be significant, $P < 0.001$ (Figure 2B).

We then isolated fibroblasts from ovarian tumours and co-cultured them with tumour cells to determine if the tumour fibroblasts behaved in a similar way to normal fibroblasts in co-culture. Co-culture of ovarian carcinoma cell lines with three different batches of fibroblasts derived from ovarian carcinomas resulted in similar structures to those described in Figure 1 and also resulted in a significant stimulation of proMMP-2 release, $P < 0.05$ (Figure 2C). The higher basal release of proMMP-2 from tumour fibroblasts resulted in a lower fold increase of proMMP-2 release compared to that found in dermal fibroblast co-cultures (Figure 2B, C).

Western blotting of protein from cultured supernatants with MMP-2 antisera confirmed the results seen with gelatin zymography (Figure 3); co-culture of dermal fibroblasts and the ovarian carcinoma cell lines enhanced the release of proMMP-2 into the culture media (two- to sixfold increase in three independent experiments). The level of TIMP-2 immunoreactivity in culture medium from co-cultures was also stimulated by a similar degree (Figure 3).

Conditioned medium from the carcinoma cell lines PEO1, PEO14 and SKOV3 did not enhance the release of proMMP-2 from dermal fibroblasts and also had no effect on fibroblast proliferation (data not shown). However, culture of dermal fibroblasts and fixed carcinoma cells resulted in an significant increase in proMMP-2 release from dermal fibroblasts, $P < 0.01$ (Figure 4). Culture of tumour fibroblasts and fixed carcinoma cells also stimulated proMMP-2 release, but fixed fibroblast cell membranes did not enhance proMMP-2 release from the carcinoma cells (data not shown).

### ProMMP-2 activation

Co-culture of fibroblasts and ovarian carcinoma cell lines did not result in significant activation of proMMP-2 (Figures 2A and 3). A trace amount of the activated 62 kDa form of MMP-2 could be detected in the medium from co-cultures, but it was < 1% of total MMP-2 activity. However, the activated form of MMP-2 is readily detected in ovarian carcinoma tissue (Naylor et al, 1994; Noel et al, 1994; Ito et al, 1995). We therefore investigated the expression of MT-MMP-type enzymes in fibroblast and carcinoma cells, and the ability of cell membranes to activate proMMP-2.

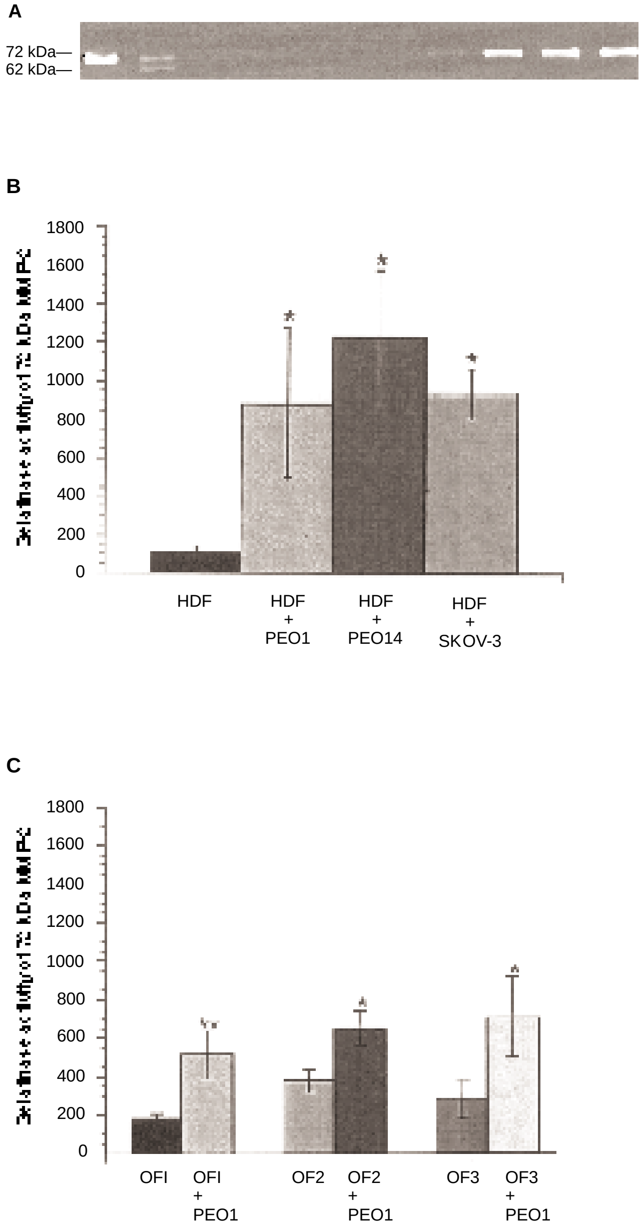

**Figure 2** Effect of co-culture of ovarian carcinoma cell lines PEO1, PEO14 and SKOV-3 with dermal or tumour fibroblasts on the release of MMP-2 into the culture medium. (**A**) Fibroblasts, carcinoma cells and carcinoma and fibroblast cells were incubated overnight in culture medium + serum. Cells were then washed and incubated for 48 h in serum-free medium. The resulting conditioned medium was analysed by gelatin zymography. ProMMP-2 standard (1), proMMP-2 and 62-kDa MMP-2 standards (2), conditioned medium from fibroblasts (3), PEO1 (4), PEO14 (5), SKOV-3 (6), fibroblasts + PEO1 (7), fibroblasts + PEO14 (8) and fibroblasts + SKOV-3 (9). These results are representative of three separate experiments. (**B**) Culture medium from dermal fibroblasts (HDF), fibroblasts + PEO1, fibroblasts + PEO14, and fibroblasts + SKOV-3, were analysed by quantitative gelatin zymography. The proMMP-2 release from the carcinoma cell lines alone was subtracted from the co-culture values. The results are means ± s.d. from four independent experiments (* represents $P < 0.001$). (**C**) Culture medium from three different batches of ovarian tumour fibroblasts (OF) with the carcinoma cell line PEO1 was analysed by quantitative zymography. The MMP-2 release from PEO1 cells alone was subtracted from the co-culture value. The results are means ± s.d. for two independent experiments. * represents $P < 0.05$ and ** $P < 0.01$

© Cancer Research Campaign 1999

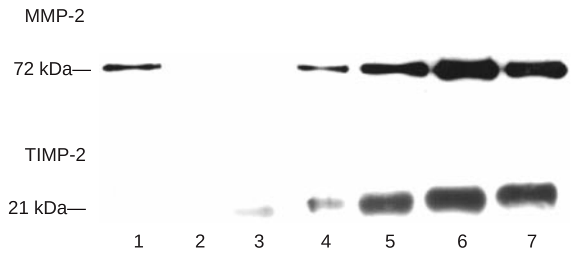

**Figure 3** Effect of co-culture of the carcinoma cell lines PEO1, PEO14 and SKOV-3 with dermal fibroblasts on the release of MMP-2 and TIMP-2 into the culture media. The culture medium was concentrated, run on an 11% SDS-polyacrylamide gel, then blotted. MMP-2 and TIMP-2 were detected by specific antisera. Conditioned medium from dermal fibroblasts (1), PEO1 (2), PEO14 (3), SKOV-3 (4), fibroblasts + PEO1 (5), fibroblasts + PEO14 (6) and fibroblasts + SKOV-3 (7). The blots are representative of three independent experiments

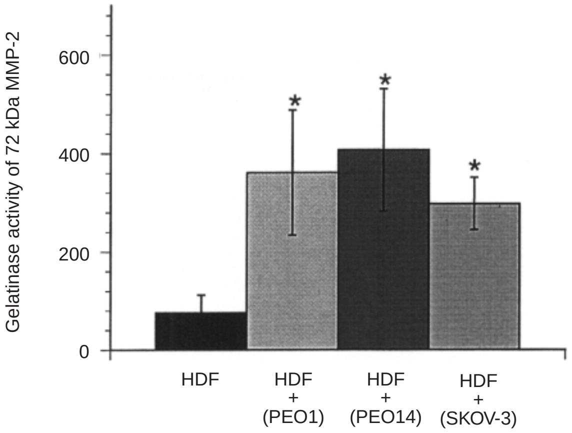

**Figure 4** Effect of methanol-fixed carcinoma cell lines on the release of proMMP-2 from fibroblasts. PEO1, PEO14 and SKOV-3 ($12.5 \times 10^4$ cells) cells were incubated overnight in culture medium + serum. The carcinoma cells were fixed by incubation in ice cold methanol for 5 min, dermal fibroblasts ($2.5 \times 10^4$ cells) were added and cultured overnight in culture medium + serum. After 24 h, the cells were washed and incubated in serum free medium for 48 h. The medium was then analysed by quantitative gelatin zymography. The results are means ± s.d. for three independent experiments done in duplicate. * represents $P < 0.01$

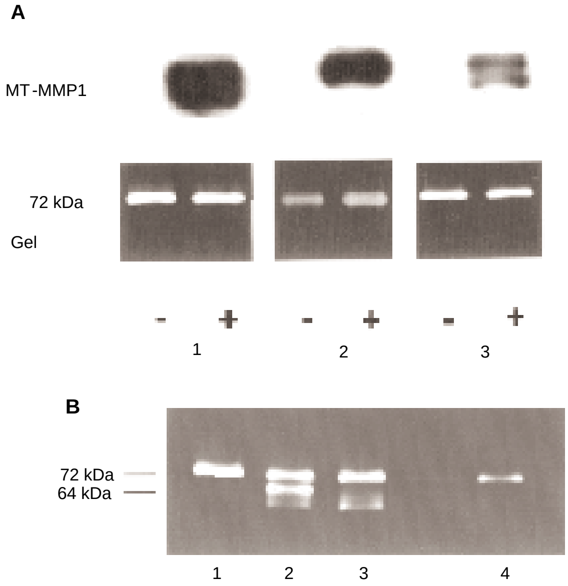

**Figure 5** (**A**) Effect of concanavalin A on MT-MMP-1 protein in membranes from ovarian tumour fibroblasts and the activation of proMMP-2. Ovarian fibroblast batches 1, 2 and 3 were treated with (+) or without (–) concanavalin A (50 μg ml$^{-1}$) for 24 h. The fibroblast membranes were extracted with 1.5% Triton X-114, the proteins in the detergent phase run on an SDS-polyacrylamide gel, blotted and MT-MMP-1 detected using specific antiserum. The culture medium from concanavalin A-treated cells was analysed by quantitative gelatin zymography. This experiment is representative of three performed. (**B**) Activation of proMMP-2 by Triton X-114 membrane protein extract of tumour fibroblasts. All incubations were for 48 h at 37°C. ProMMP-2 with Triton X-114 (1), membrane protein extract with proMMP-2 (2), membrane protein extract with proMMP-2 and BB2116 (30 μM) (3), membrane protein extract plus proMMP-2 complexed with TIMP-2 (4). This experiment is representative of three performed

### *Membrane-bound metalloproteinase activators of proMMP-2*

Using RT-PCR we found that fibroblasts (both dermal and tumour), PEO14, SKOV3, but not PEO1, ovarian cells all expressed MT-MMP-1 mRNA. RT-PCR analysis of MT-MMP-1, -2, -3, and -4 revealed no qualitative difference in the expression profile for the dermal and tumour fibroblasts. The fibroblasts, PEO14 and SKOV3 cells also had a low level of MT-MMP-1 protein in their membranes. To elevate the level of membrane MT-MMP-1 (both the 63 and the activated 60 kDa forms) we treated tumour fibroblasts with 50 μg ml$^{-1}$ concanavalin A (Lohi et al, 1996). However, this did not result in the activation of extracellular proMMP-2 by the tumour fibroblasts (Figure 5A).

Incubation of proMMP-2 with a membrane protein extract of tumour fibroblasts (detergent phase from Triton X-114-treated tumour fibroblasts) converted the 72 kDa proMMP-2 to the 64 kDa form of MMP-2. This activation of MMP-2 was blocked by an inhibitor of matrix metalloproteinases, BB2116 (30 μM), suggesting that a MMP-type enzyme was involved in proMMP-2 processing (Figure 5B). As the tumour fibroblasts released TIMP-2 as well as proMMP-2 (data not shown), we incubated MMP-2 complexed with TIMP-2 with the detergent phase from Triton X-114-extracted tumour fibroblasts. No activation of proMMP-2 was seen under these conditions (Figure 5B). The detergent phase from Triton X-114-extracted PEO14 and SKOV3, but not PEO1, cells was also able to activate proMMP-2 and this activation of MMP-2 was blocked by 30 μM BB2116 (data not shown).

Taken together, these results suggest that MT-MMP-type enzymes in the membrane of both tumour cells and fibroblasts can activate proMMP-2, but in co-culture their action is blocked, most likely by TIMP-2. We therefore investigated factors in the extracellular environment of tumours which could stimulate proMMP-2 activation and account for the presence of active MMP-2 in ovarian cancer biopsies. One potential candidate was collagen I, which is a major component of ovarian carcinoma tissue (Zhu et al, 1994). Previous studies showed that culture of normal fibroblasts in collagen gels results in proMMP-2 activation (Azzam and Thompson, 1992; Seltzer et al, 1994; Gilles et al, 1997).

### *Collagen I treatment of fibroblasts*

Treatment of normal dermal fibroblasts with soluble collagen I (100 μg ml$^{-1}$) did not increase activation of proMMP-2 (Figure 6A). However, incubation of three different batches of

 © Cancer Research Campaign 1999

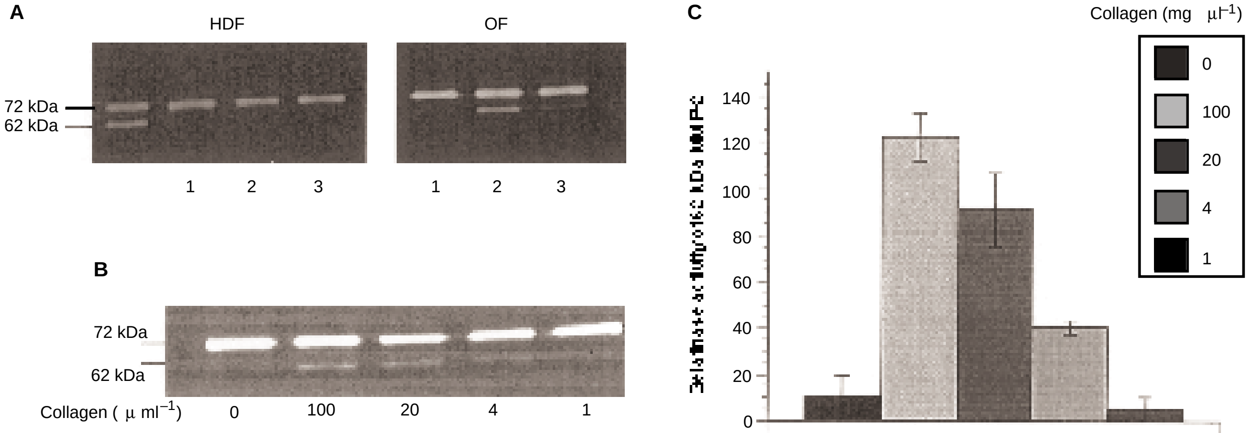

**Figure 6** (**A**) Zymogram of medium from either dermal (HDF) or ovarian carcinoma-derived fibroblasts (OF) treated with collagen I or BB2116. Cells were treated with serum-free medium (1), plus 100 µg collagen I per ml (2), or 100 µg collagen I per ml and 30 µM BB2116 (3) for 24 h at 37°C. The experiment is typical of three performed. (**B**) Zymogram of medium from ovarian-derived fibroblasts treated with different amounts of collagen I. (**C**) Effect of different collagen I concentrations on the induction of proMMP-2 activation in ovarian tumour fibroblasts. Cells were treated with 100, 20, 4 and 1 µg ml$^{-1}$ of collagen I for 24 h. The results are means ± s.d. for two independent experiments done in duplicate

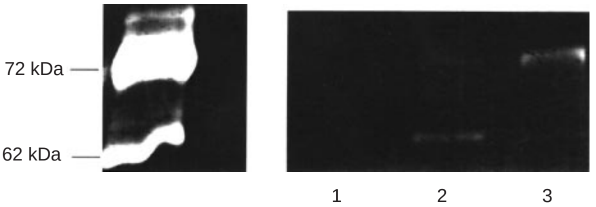

**Figure 7** Detection of gelatinase activity in the Triton X-114 detergent phase from fibroblasts treated with collagen I or concanavalin A. Ovarian fibroblasts were treated with serum-free medium (1), 100 µg collagen I per ml (2) or 50 µg ml$^{-1}$ concanavalin A (3). After incubation with 1.5% Triton X-114 for 1 h at 4°C the detergent phase was treated with gelatin-coated agarose beads. MMPs attached to the beads were removed by sample buffer and analysed by quantitative gelatin zymography. The experiment is typical of three performed

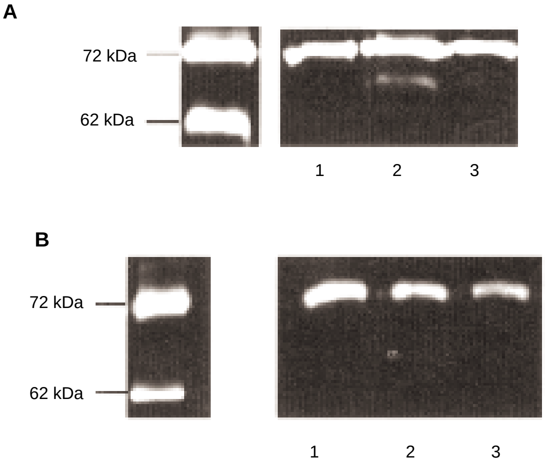

**Figure 8** Effect of anti-$\beta_1$ integrin antibody on proMMP-2 activation by tumour-derived fibroblasts. (**A**) Ovarian fibroblasts were treated with serum-free medium (1) anti-$\beta_1$ integrin antibody (2) or anti-$\beta_1$ integrin antibody + BB2116 (3). (**B**) Ovarian fibroblasts were treated with serum-free medium (1) anti-$\beta_1$ integrin antibody (2) or anti-$\beta_1$ integrin antibody + BB2116 (3). The culture medium was analysed by quantitative gelatin zymography. The experiment is typical of three completed experiments tumour fibroblasts with collagen I (100 µg ml$^{-1}$) resulted in the appearance of the fully activated 62 kDa form of MMP-2 in the cell culture medium (Figure 6A shows a typical result). A faint gelatinolytic band of 64 kDa could also be detected between the 72 and 62 kDa forms of MMP-2 (Figure 6A). BB-2116 (30 µM) blocked the activation of proMMP-2 by tumour fibroblasts in the presence of collagen I (Figure 6A).

The amount of 62 kDa MMP-2 detected in the culture medium of collagen I-treated cells was increased by 10 ± 2-fold in three experiments. This activated form of MMP-2 could also be detected by Western blotting with specific MMP-2 antiserum (data not shown). The effects of collagen I (4–100 µg ml$^{-1}$) on proMMP-2 activation were dose-dependent (Figure 6B). The response of tumour fibroblasts to collagen I was only present for the first few passages after isolation (data not shown).

### *The association of active MMP-2 with cell membranes*

We next looked for the presence of active MMP-2 in the membranes of collagen I and concanavalin A-treated tumour fibroblasts. Tumour fibroblasts were incubated with medium alone, collagen I or concanavalin A. Fibroblasts were treated with Triton X-114 and the detergent phase (containing membrane proteins) was analysed for gelatinase activity. The detergent phase from fibroblasts treated with medium alone contained no gelatinase activity. However, the detergent phase from collagen I-treated cells contained a 62 kDa band, whereas the detergent phase from concanavalin A-treated cells contained only a 72 kDa gelatinase band (Figure 7).

### *Integrin-mediated activation of MMP-2*

Collagen I can interact with integrin collagen receptors such as $\alpha_2\beta_1$ and $\alpha_1\beta_1$. Treatment of tumour fibroblasts with specific anti-$\beta_1$ integrin antibody (10 µg ml$^{-1}$) also resulted in the activation of proMMP-2 but only the 64 kDa form of MMP-2 could be detected; BB-2116 (30 µM) blocked proMMP-2 activation by both collagen 1 and anti-$\beta_1$ integrin antibody (Figure 8). Treatment of normal dermal fibroblasts with the anti-$\beta_1$ integrin did not result in proMMP-2 activation (Figure 8).

*© Cancer Research Campaign 1999*

### DISCUSSION

In this study, an in vitro co-culture system was used to investigate the mechanisms by which ovarian carcinoma cells stimulate MMP-2 release and activation by fibroblasts. Culture of ovarian carcinoma cells with normal dermal fibroblasts or ovarian tumour fibroblasts resulted in a stimulation of proMMP-2 release into the medium. The stimulation of proMMP-2 release, seen when tumour fibroblasts were cultured with ovarian cancer cells, was lower than that seen with dermal fibroblasts, probably because of higher basal release of proMMP-2 from tumour fibroblasts.

In co-cultures of normal fibroblasts and carcinoma cell lines, TIMP-2 release was also enhanced. The stimulation of proMMP-2 and TIMP-2 release from co-cultures may suggest that the release of these proteins is co-ordinately regulated. This observation is also consistent with the lack of proMMP-2 activation found in co-cultures since TIMP-2 inhibits MT-MMP-1 mediated MMP-2 activation.

Cultured ovarian carcinoma cells or cell lines release both MMP-2 and TIMP-2 (Moser et al, 1994). On the basis of in vitro studies it has been proposed that ovarian carcinoma cells may produce MMP-2 in vivo (Fishman et al, 1997; Westerlund et al, 1997). However, in ovarian cancer biopsies, MMP-2 and TIMP-2 mRNAs localize to the stroma. Epithelial carcinoma cell production of these molecules was not detected (Naylor et al, 1994). This suggests that production of proMMP-2 and TIMP-2 by cultured carcinoma cells may be an adaptation to in vitro culture conditions.

Culture of fibroblasts with fixed carcinoma cells indicates that cell–cell contact plays an important part in the enhancement of proMMP-2 release seen in co-cultures. This is in agreement with the in vivo data where only the fibroblasts close to, or in contact with, carcinoma cells have elevated mRNA for MMP-2 and TIMP-2 (Naylor et al, 1994). The nature of the membrane factor(s) that stimulates proMMP-2 and TIMP-2 release is unknown. EMMPRIN has been proposed as a regulator of MMP-2 release from fibroblasts (Kataoka et al, 1993) and, although this protein could be detected in PEO1 and PEO14 membranes, it could not be detected in SKOV3 cell membranes, yet all three stimulated proMMP-2 release (data not shown).

Both tumour fibroblasts and carcinoma cells express MT-MMP-1 protein (Fishman et al, 1997) and we confirmed this in our study. MT-MMP-1 mRNA is expressed in stromal fibroblast-like cells close to tumour cells in tumour tissue (Okada et al, 1995; Ohtani et al, 1996; Ueno et al, 1997). It has been proposed that MT-MMP-1 is responsible for the activation of proMMP-2 in ovarian carcinoma tissue (Fishman et al, 1997). However, co-culture of ovarian carcinoma cells and normal or tumour fibroblasts did not result in the activation of MMP-2, probably because extracellular TIMP-2 blocked proMMP-2 activation. Even when the amount of MT-MMP-1 in tumour fibroblasts was increased by concanavalin A treatment, no significant activation of MMP-2 could be seen, in agreement with a recent study on primary human fibroblasts (Lohi et al, 1996). Hence the expression of MT-MMP-1 protein does not always correlate with MMP-2 activation. The ovarian carcinoma microenvironment is rich in TIMPs (Naylor et al, 1994; Tamamkoshi et al, 1995; Kikkawa et al, 1997) and these inhibitors might be predicted to block the activity of MT-MMP-type enzymes.

We have found that levels of collagen I as low as 4 μg $ml^{-1}$ can stimulate the activation of proMMP-2 by tumour fibroblasts, even in the presence of TIMPs. Normal fibroblasts did not display this responsiveness to similar doses of collagen I. Incubation of normal fibroblasts or invasive breast cancer cells in collagen I gels (containing 0.5–1 mg $ml^{-1}$ of collagen I), results in gross morphological changes and the intracellular activation of proMMP-2 by a metalloproteinase-like enzyme, but no change in TIMP-2 release (Seltzer et al, 1994; Gilles et al, 1997; Lee et al, 1997). Gilles et al (1997) also reported that three dimensional collagen gel culture up-regulated MT-MMP-1. However, at the lower collagen concentrations used in our study, the tumour-derived fibroblasts seem to exhibit an enhanced responsiveness to collagen.

It is likely that collagen I is acting through the $\alpha_2\beta_1$ or $\alpha_1\beta_1$ receptors on the tumour-derived fibroblasts to stimulate proMMP-2 activation to the 62 kDa form of MMP-2. Anti-$\beta_1$ integrin antibody induced tumour, but not normal, fibroblasts to activate proMMP-2 into the 64 kDa form of MMP-2. This result suggests that the $\beta_1$ integrin subunit is involved in the differential response of tumour fibroblasts to collagen I; this could occur by an up-regulation of the $\beta_1$ subunit and/or integrins or an enhanced affinity of $\beta_1$ integrin for collagen I. It is of interest that treatment of rhabdomyosarcoma cells with anti-$\alpha_3$ and anti-$\alpha_2$ integrin antibodies increased their ability to activate MMP-2, and also increased proMMP-2 secretion and invasiveness through matrigel (Kubota et al, 1997).

There are at least three possible mechanisms by which collagen 1 treatment leads to proMMP-2 activation: collagen I could induce the expression of a metalloproteinase or metalloproteinase activator, it may reduce TIMP-2 levels, or it may relocalize MT-MMP-1 to specific cellular sites. At the site of invasion, $\beta_1$ integrin may possibly interact with MT-MMP-1. We also have preliminary data that collagen 1 does not up-regulate metalloprotease activity in tumour fibroblasts, again favouring the third hypothesis.

In conclusion, we have found that fibroblast–carcinoma cell interaction in vitro results in the stimulation of proMMP-2 and TIMP-2 release by fibroblasts. This corresponds to our in vivo observations of MMP-2 and TIMP-2 mRNA expression and localization (Naylor et al, 1994). We have also found that ovarian tumour-derived fibroblasts have a novel responsiveness to collagen I with respect to proMMP-2 activation. Collagen I stimulation of proMMP-2 activation by tumour-derived fibroblasts appears to involve the ligation of the $\beta_1$ integrin and provides a mechanism by which ovarian tumours could activate MMP-2, despite the presence of TIMPs. The binding of this activated MMP-2 by receptors in the invadopodia of invasive cells, provides a temporal window of opportunity for the utilization of active MMP-2 in cell migration and invasion.

### ACKNOWLEDGEMENTS

The authors wish to thank Thomas Leber of the Biological Therapy Laboratory for helpful discussion.

### REFERENCES

Afzal S, Lalani E-L, Foulkes WD, Boyce B, Tickle S, Cardillo M-R, Baker T, Pignatelli M and Stamp GWH (1996) Matrix metalloproteinase-2 and tissue inhibitor of metalloproteinase-2 expression and synthetic matrix metalloproteinase-2 inhibitor binding in ovarian carcinomas and tumour cell lines. *Lab Invest* **2**: 406–421

Atkinson SJ, Crabbe T, Cowell S, Ward R, Butler MJ, Sato H, Seiki M, Reynolds JJ and Mutphy G (1995) Intermolecular autolytic cleavage can contribute to the

© Cancer Research Campaign 1999

activation of progelatinase A by cell membranes. *J Biol Chem* **270**: 30479–30485

Autio-Harmainen H, Karttunen T, Hurskainen T, Hoyhtya M and Kauppila A (1993) Expression of 72 kilodalton type IV collagenase (gelatinase A) in benign and malignant ovarian tumours. *Lab Invest* **69**: 312–321

Azzam HS and Thompson EW (1992) Collagen-induced activation of the $M_r$ 72 type IV collagenase in normal and malignant human fibroblastoid cells. *Cancer Res* **52**: 4540–4544

Baramova EN, Bajou K, Remacle A, Líhoir C, Krell HW, Weidle UH, Noel A and Foidart JM (1997) Involvement of PA/plasmin system in the processing of pro-MMP-9 and in the second step of proMMP-2 activation. *FEBS Letts* **405**: 157–162

Birkedal-Hansen H (1995) Proteolytic remodeling of extracellular matrix. *Curr Opin Cell Biol* **7**: 728–735

Bordier C (1981) Phase separation of integral membrane proteins in Triton X-114 solution. *J Biol Chem* **256**: 1604–1607

Campo E, Merino MJ, Tavassoli FA, Charonis AS and Stetler-Stevenson WG (1992) Evaluation of basement membrane components and the 72 kDa type IV collagenase in serous tumours of the ovary. *Am J Surg Pathol* **16**: 500–507

Fishman DA, Bafetti LM and Stack MS (1997) Membrane-type matrix metalloproteinase expression and matrix metalloproteinase-2 activation in primary human ovarian epithelial carcinoma cells. *Inv Metastasis* **16**: 150–159

Garzetti G, Ciavattini A, Lucarini G, Goteri G, Nictolis MD, Garbisa S, Masiero L, Romanini C and Graziella B (1995) Tissue and serum metalloproteinase (MMP-2) expression in advanced ovarian serous cystodenocarcinomas: clinical and prognostic implications. *Anticancer Res* **15**: 2799–2804

Gilles C, Polette M, Seiki M, Birembaut P and Thompson EW (1997) Implication of collagen type-1 induced membrane-type 1-matrox metalloproteinase expression and matrix metalloproteinase-2 activation in the metastatic progression of breast carcinoma. *Lab Invest* **76**: 651–660

Greene J, Wang M, Liu YI, Raymond LA, Rosen C and Shi YI (1996) Molecular cloning and characterization of human tissue inhibitor of metalloproteinase 4. *J Biol Chem* **271**: 30375–30378

Ito A, Nakajima S, Sasaguri Y, Nagase H and Mori Y (1995) Co-culture of human breast adenocarcinoma MCF-7 cells and human dermal fibroblasts enhances the production of matrix metalloproteinases 1, 2 and 3 in fibroblasts. *Br J Cancer* **71**: 1039–1045

Kataoka H, Decastro R, Zucker S and Biswas C (1993) Tumour cell-derived collagenase-stimulatory factor increases expression of interstitial collagenase, stromeolysin, and 72 kDa gelatinase. *Cancer Res* **53**: 3154–3158

Kikkawa F, Tamakoshi K, Nawa K, Shibata K, Yamagata S, Yamagata T and Suganuma N (1997) Positive correlation between inhibitors of matrix metalloproteinase 1 and matrix metalloproteinases in malignant ovarian tumour tissues. *Cancer Letts* **120**: 109–115

Kolenbrock H, Hecker-Kia A, Orgel D, Ulbrich N and Will H (1997) Activation of progelatinase A and progelatinase A/TIMP-2 complex by membrane type 2-matrix metalloproteinase. *J Biol Chem* **378**: 71–76

Kubota S, Ito H and Seyama Y (1997) Anti-alpha3 integrin antibody induces the activated form of matrix metalloprotease-2 (MMP-2) with concomitant stimulation of invasion through matrigel by human rhabdomyosarcoma cells. *Int J Cancer* **70**: 106–111

Kuwashima Y, Uehara T, Kurosumi M, Kishi K, Shironizu K, Matsuzawa M and Takayama S (1995) Basement membrane status I undifferentiated carcinomas of the ovary: immunohistochemical distribution of type IV collagen and laminin. *Eur J Gynaecol Oncol* **16**: 181–186

Leber TM and Balkwill FR (1997) Zymography: a single-step staining method for quantitation of proteolytic activity on substrate gels. *Anal Biochem* **249**: 24–28

Lee AY, Akers KT, Collier M, Li L, Eisen AZ and Louise-Seltzer J (1997) Intracellular activation of gelatinase A (72-kDa type IV collagenase) by normal fibroblasts. *Proc Natl Acad Sci USA* **94**: 4424–4429

Lewalle JM, Munaut C, Pichot B, Cataldo D, Baramova E and Foidart JM (1995) Plasma membrane-dependent activation of gelatinase A in human vascular endothelial cells. *J Cell Physiol* **165**: 475–483

Liabakk N-B, Talbot I, Smith RA, Wilkinson K and Balkwil FB (1996) Matrix metalloprotease 2 (MMP-2) and matrix metalloprotease 9 (MMP-9) type IV collagenases in colorectal cancer. *Cancer Res* **56**: 190–196

Liotta LA, Steeg PS and Steteler-Stevenson WG (1991) Cancer metastasis and angiogenesis: an imbalance of positive and negative regulation. *Cancer Res* **64**: 327–336

Lohi J, Lehti K, Westermarck J, Kahari V-M and Keski-Oja J (1996) Regulation of membrane-type matrix metalloprotease-1 expression by growth factors and phorbol 12-myristate 13-acetate. *Eur J Biochem* **239**: 239–247

Moser TL, Young TN, Rodriguez GC, Pizzo SV, Bast RC and Stack MS (1994) Secretion of extracellular matrix-degrading proteinases is increased in epithelial ovarian carcinoma. *Int J Cancer* **56**: 552–559

Naylor MS, Stamp GW, Davies BD and Balkwill FR (1994) Expression and activity of MMPs and their regulators in ovarian cancer. *Int J Cancer* **58**: 50–56

Niedbala MJ, Crickard K and Bernacki RJ (1987) In vitro degradation of extracellular matrix by human ovarian carcinoma cells. *Clin Exp Metastasis* **5**: 181–197

Noel A, Munaut C, Boulvain A, Calberg-Bacq CM, Nusgens B, Lapiere CM and Foidart JM (1992) Modulation of collagen and fibronectin synthesis in fibroblasts by normal and malignant cells. *J Cell Biochem* **48**: 150–161

Noel A, Polette M, Lewalle J-M, Munaut C, Emonard HP, Birembaut P and Foidart J-M (1994) Co-ordinate enhancement of gelatinase A mRNA and activity levels in human fibroblasts in response to breast-adenocarcinoma cells. *Int J Cancer* **56**: 331–336

Ohtani H, Motohashi H, Sato H, Seiki M and Nagura H (1996) Dual overexpression of membrane-type metalloproteinase-1 in cancer and stromal cells in human gastrointestinal carcinoma revealed by in situ hybridization and immunoelectron microscopy. *Int J Cancer* **68**: 565–570

Okada A, Bellocq J-P, Rouyer N, Chenard M-P, Rio M-C, Chambon P and Basset P (1995) Membrane-type metalloproteinase (MT-MMP) gene is expressed in stromal cells of human colon, breast, and head and neck carcinomas. *Proc Natl Acad Sci USA* **92**: 2730–2734

Ray JM and Stetler-Stevenson WG (1995) Gelatinase A activity directly modulates melanoma cell adhesion and spreading. *EMBO J* **14**: 908–917

Sato H, Takino T, Okada O, Kada O, Cao J, Shinagawa A, Yamamoto E and Seiki M (1994) A matrix metalloproteinase expressed on the surface of invasive tumour cells. *Nature* **370**: 61–65

Seltzer JL, Lee A-Y, Akers KT, Sudbeck B, Eileen A, Southon EA, Wayner EA and Eisen AZ (1994) Activation of 72-kDa collagenase/gelatinase by normal fibroblasts in collagen lattices is mediated by integrin receptors but is not related to lattice contraction. *Exp Cell Res* **213**: 365–374

Shofuda K, Yasumitsu H, Nishihashi A, Miki K and Miyazaki K (1997) Expression of three matrix metalloproteinases (MT-MMPs) in rat vascular smooth muscle cells and characterization of MT3-MMPs with and without transmembrane domain. *J Biol Chem* **272**: 9749–9754

Stetler-Stevenson WG, Krutzsch HC, Wacher MP, Maraguliies IMK and Liotta IA (1989*a*) The activation of human type IV collagenase proenzyme: sequence identification of the major conversion product following organo-mecurial activation. *J Biol Chem* **264**: 1353–1360

Stetler-Stevenson WG, Krutzsch HC and Liotta IA (1989*b*) Tissue inhibitors of metalloproteinase (TIMP-2): a new member of the metalloproteinase inhibitor family. *J Biol Chem* **264**: 17374–17378

Strongin AY, Cillier I, Bannikov G, Marmer BL, Grant GA and Goldberg GI (1995) Mechanism of cell surface activation of 72 kDa type IV collagenase *J Biol Chem* **270**: 5331–5338

Tamakoshi K, Kikkawa F, Nawa A, Ishikawa H, Mizuno K, Tamakoshi A, Yamagata S, Suganuma N and Tomoda Y (1995) Characterisation of extracellular matrix-degrading proteinase and its inhibitor in gynecologic cancer tissues with clinically different metastatic form. *Cancer* **76**: 2565–2571

Westerlund A, Hujanen E, Puistola U and Turpeenniemi-Hujanen T (1997) Fibroblasts stimulate human ovarian cancer cell invasion and expression of 72-kDa gelatinase A (MMP-2). *Gynecol Oncol* **67**: 76–82

Ueno H, Nakamura H, Inoue M, Imai M, Imai K, Noguchi M, Sato H, Seiki M and Okada Y (1997) Expression and tissue localization of membrane-types 1, 2 and 3 matrix metalloproteinases in human invasive breast carcinomas. *Cancer Res* **57**: 2055–2060

Zhu G-G, Risteli L, Risteli J, Kauppila M and Stenback F (1994) Immunohistochemical study of type I collagen and type I pH-collagen in benign and malignant neoplasms. *Cancer* **75**: 1010–1017

© Cancer Research Campaign 1999
